# Correlation between retinal vessel rarefaction and psychometric measures in an older Southern Italian population

**DOI:** 10.3389/fnagi.2022.999796

**Published:** 2022-09-23

**Authors:** Gianluigi Giuliani, Giancarlo Sborgia, Alfredo Niro, Fabio Castellana, Luisa Lampignano, Pasquale Puzo, Angelo Pascale, Valentina Pastore, Rosa Buonamassa, Roberta Galati, Marco Bordinone, Flavio Cassano, Arcangelo Clemente, Luca Landini, Giacomo Scotti, Marida Gaudiomonte, Antonella Guglielmi, Roberto Semeraro, Michele Santoro, Giovanni Alessio, Rodolfo Sardone, Francesco Boscia

**Affiliations:** ^1^Department of Medical Science, Neuroscience and Sense Organs, Eye Clinic, University of Bari, Bari, Italy; ^2^Eye Clinic, Hospital “SS Annunziata,” ASL Taranto, Taranto, Italy; ^3^Unit of Research Methodology and Data Sciences for Population Health, “Salus in Apulia Study,” National Institute of Gastroenterology “Saverio de Bellis,” Research Hospital, Bari, Italy

**Keywords:** retina, imaging, older (diseased) population, cognitive function, vessel density

## Abstract

**Objective:**

To explore the linear association between inner retinal layers thickness and macular capillary density compared to variations of global cognition evaluated by psychometric measures in a cohort of Mediterranean subjects aged 65+ years.

**Materials and methods:**

We performed a cross-sectional analysis of 574 participants aged 65 years+ drawn from a population-based Southern Italian study. All subjects underwent neurological evaluations, including global cognitive screening, the Mini-Mental State Examination (MMSE) and frontal assessment battery (FAB), together with an ophthalmic examination including optical coherence tomography (OCT) and OCT-Angiography. We assessed the average thickness of the ganglion cell complex (GCC) and the retinal nerve fiber layer (RNFL), the foveal avascular zone area, and vascular density (VD) of superficial (SVD) and deep (DVD) capillary plexi at the foveal and parafoveal area. Linear regression was applied to assess associations of ocular measurements with MMSE and FAB scores.

**Results:**

In the linear regression model, foveal DVD (beta = 0.01, 95% CI:0.004–0.052), whole DVD (beta = 0.04, 95% CI:0.02–0.08), and whole SVD (beta = 0.04, 95% CI:0.02–0.07) showed a positive association with MMSE. In addition, foveal SVD (beta = 0.01, 95% CI:0.003–0.05) and whole SVD (beta = 0.03, 95% CI:0.004–0.08) were positively associated with the FAB score. We found no further significant association between the MMSE score or the FAB score and the average thickness of the GCC and RNFL, and FAZ area.

**Conclusion:**

A direct linear association between the VD of the macular capillary plexi with global and frontal cognitive functions was observed in elderly subjects.

## Introduction

As the world population ages, mild cognitive impairment (MCI) and dementia are taking on an ever growing social importance. Cognitive impairment is the most evident sign of all dementias including Alzheimer’s disease (AD), known to account for between 60 and 80% of all cases (Hebert et al., 2013). According to current knowledge, in the USA alone, 5.8 million people are affected by cognitive impairment and this figure is expected to rise to 13.8 million by 2050 (Hebert et al., 2013).

Aging and cardiovascular conditions have been suggested to drive chronic cerebral hypoperfusion (CCH) leading to neurodegenerative processes (Scheffer et al., 2021). Indeed, cerebral vascular and microvascular remodeling has been described in patients with cognitive impairment (Buée et al., 1994, 1997; Farkas and Luiten, 2001; O’Brien et al., 2003; Thompson and Hakim, 2009).

In recent decades, much of the research has been focused on recognizing biomarkers enabling diagnosis of the disease in the early stages (Shaw et al., 2007; Becker et al., 2008; Holtzman et al., 2011; Sperling et al., 2014). Indeed, neuropathological changes in the central nervous system are known to occur many years before the clinical onset of severe or MCI (Shaw et al., 2007; Becker et al., 2008; Holtzman et al., 2011; Sperling et al., 2014).

Embryologically, anatomically, and physiologically, the retina is an extension of the central nervous system and hence offers a privileged observational viewpoint (Byerly and Blackshaw, 2009; London et al., 2013; Trost et al., 2016; Vecino et al., 2016).

In this context, optical coherence tomography (OCT) scans previously confirmed thinning of the neuroretinal layers, including the ganglion cell complex (GCC) and, in particular, the retinal nerve fiber layer (RNFL), in AD (Chan et al., 2019) and MCI patients (Mejia-Vergara et al., 2020) as compared to age-matched control subjects. Also retinal microvascular changes, visualized non-invasively using fundoscopy or photographs of the retina, have been associated with cognitive decline and brain changes related to aging and vascular disease (Wong et al., 2002; Baker et al., 2007; Ding et al., 2008; Heringa et al., 2013). Optical coherence tomography angiography (OCT-A) is an innovative extension of OCT technology. It provides non-invasive depth-resolved visualization of the retinal microvasculature, using phase or amplitude decorrelation to identify the motion contrast of blood flow (Spaide et al., 2015; Kashani et al., 2017). It has been successfully used in several studies to explore the predictive power of retinal vessel features for neurodegenerative diseases (Bulut et al., 2018; O’Bryhim et al., 2018; Zhang et al., 2019), revealing a reduction of vascular density (VD) at the macula in patients with MCI and AD (Bulut et al., 2018; Jiang et al., 2018; Yoon et al., 2019).

Together with personal history, physical examination and laboratory tests, psychometric measures, such as the Mini-Mental State Examination (MMSE), Alzheimer’s disease assessment scale- (ADAS-Cog), clinical dementia rating (CDR) score, and frontal assessment battery (FAB), are used nowadays to test a range of everyday cognitive abilities that can reveal cognitive impairment.

In AD but not in MCI patients, multiple linear regression models showed a strong association between overall RNFL thickness and the MMSE score (Ascaso et al., 2014). Moreover, Shen et al. (2013) observed that RNFL thinning could have a predictive role in cognitive decline.

The aim of this study was to explore the linear association between inner retinal layers thinning and macular capillary density reductions and variations of global cognition measures, including the MMSE and FAB, in a cross-sectional study of Mediterranean subjects aged 65 + years.

## Materials and methods

Participants were recruited from the Salus in Apulia Study, a population-based study on aging conducted on subjects aged over 65 years, resident in Castellana Grotte, a town located near Bari, Puglia in the South-east of Italy. The final sample frame was the Castellana Grotte electoral list on 31 December 2014, including 19,675 subjects, 4,008 aged 65 years or older. The Salus in Apulia Study has focused on the impact of nutrition, frailty, and age-related sensory impairments as predictors of common neurodegenerative and psychiatric diseases in older age. Detailed methodology of the original study is reported elsewhere (Tortelli et al., 2017). We collaborated with general practitioners who invited older subjects, previously selected with the support of the city census office, to participate in the study. Subjects enrolled in the SALUS study underwent several multi-specialist visits, including neurological and neuropsychological assessments and a complete ophthalmological evaluation. In particular, in this study we analyzed data on subjects who underwent OCT and OCT-A examinations. Prior approval of the study was obtained from the Institutional Review Board of the “National Institute of Gastroenterology “S. De Bellis” (Approval Code: 68/CE De Bellis; Approval Date: 9 April 2019). Written informed consent was obtained from all the participants in this study, all community-dwelling older adults.

The present study adhered to the “Standards for Reporting Diagnostic Accuracy Studies” (STARD) guidelines,^1^ the “Strengthening the Reporting of Observational Studies in Epidemiology” (STROBE) guidelines,^2^ and was conducted in accordance with the 1975 Declaration of Helsinki.

### Ophthalmological assessment

Each participant underwent best-corrected visual acuity (BCVA) determination of each eye. BCVA was recorded as Snellen visual acuity and converted to the logarithm of minimal angle of resolution (LogMar) units for statistical analysis. Patients underwent slit-lamp biomicroscopy and intraocular pressure (IOP) measurement using a Goldmann-type applanation tonometer (Perkins MK2 Handheld Tonometer, Clement-Clarke Haag-Streit, Essex, UK). We performed funduscopy and then OCT and OCT-A using AngioVue OCT-Angiography (Optovue RTVue XR 100 AVANTI, Optovue, Inc., Fremont, CA, United States). The OCT-A machine captures two consecutive B-scans (M-B frame), each containing 304 A-scans with an A-scan rate of 70,000 scans per second, using a light source with a bandwidth of 45 nm centered on 840 nm. Split-spectrum amplitude-decorrelation angiography (SSADA) then extracts blood flow information by quantifying the decorrelation value, which represents differences in signal intensity between consecutive B-scans of the same location on the retina. OCT-A also analyzes the retinal structure, so multiple retinal layers can be identified and the vasculature in the corresponding layers can be segmented. OCT segmentation was performed using the AngioVue module with Optovue RTVue AVANTI software (version 2015.100.0.35, Optovue, Inc., Fremont, CA, United States). The mode was set at Angio Retina (3 mm× 3 mm) and Angio Disc (4.5 mm × 4.5 mm). RTvue software includes Optovue Motion Correction Technology (MCT) and 3D Projection Artifact Technology. The software provides the signal strength index (SSI), which represents the scan’s reflectance signal strength, and a quality index (Q-score), which represents the overall quality of the image, taking into account factors like SSI and motion artifacts (Kashani et al., 2017). In the present study, we only included images with a Q-score of 6 or above, an SSI above 70, and without motion or shadow artifacts. The examinations were performed blinded by trained ophthalmologists.

The vessel density (VD,%), defined as the percentage area occupied by the vessels in the corresponding region, was automatically measured by the built-in OCT device software.

**FIGURE 1:**
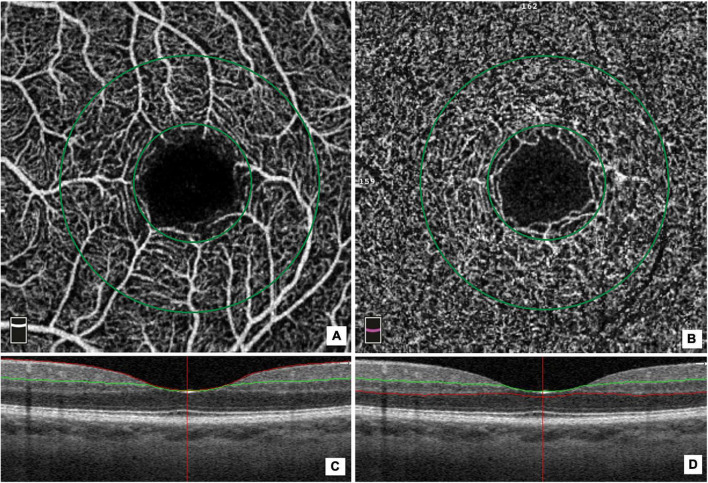
Optical coherence tomography (OCT) angiographic images of the macular region and corresponding structural OCT scans. The macular vessel density measurement included measurements of the superficial **(A)** and deep vascular **(B)** plexi in an area measuring 3 mm× 3mm. The macular area was divided into a foveal and a parafoveal area between two concentric circles with a 1 and 3 mm diameter, respectively **(A,B)**. The colored lines (red and green) in horizontal OCT B-scans show segmentation lines defining the different depths in the retinal tissue. The superficial capillary plexus is segmented from approximately 3 μm below the inner limiting membrane to 15 μm below the inner plexiform layer (IPL) **(C)**. The deep capillary plexus is segmented from 15 μm below the IPL to 70 μm below the IPL **(D)**.

The OCT angiograms centered on the fovea were automatically segmented to define the superficial plexus, from 3 μm below the internal limiting membrane to 15 μm below the inner plexiform layer (IPL), and the deep plexus, from 15 to 70 μm below the inner plexi-form layer. The VD at each macular plexus, and superficial VD (SVD) and deep VD (DVD), were calculated for the whole 3-mm circle area centered on the fovea (whole retina), for the area between the outer 3-mm circle and the inner 1-mm circle (parafoveal quadrant), and for the area inside the central 1-mm circle (foveal quadrant) (Figure 1).

Measurement of the foveal avascular zone (FAZ, mm^2^) at the deep capillary plexus (image in Supplementary Figure 1) was performed as described in detail elsewhere (Shahlaee et al., 2016).

The average thickness (μm) of the GCC, composed of the thickness of the RNFL, ganglion cell layer (GCL), and IPL, at the macular area, and, separately, of the RNFL, were measured at the same time using the same OCT system (image in Supplementary Figure 2).

Ocular exclusion criteria for all study participants included an IOP > 22 mmHg, a history of glaucoma, optic neuropathies, demyelinating disorders, retinal diseases including macular degeneration, diabetic or hypertensive retinopathy, epiretinal membrane, retinal detachment, an obvious media opacity reducing visual acuity below 1 LogMar and interfering with the OCT and OCT-A analysis, a refractive error of 6 diopters or more, an intraocular surgery performed in the previous 6 months or ocular trauma.

Data export was performed using the GreatAGEstudy App (Phronema srl, Bari, Italy), a software designed specifically to support the ancillary study of the Salus in Apulia Study, denominated the GreatAGE study.

### Cognitive assessment

A licensed neurologist performed a standard neurological examination exploring perception, deambulation, cranial nerves, motor function (muscle tone, upright posture, and tropism), pathological gestures, sensory function, cerebellar and sphincter functions, deep tendon reflexes, and signs of diffuse cerebral distress.

Cognitive mental status was assessed with the MMSE, which consists of eleven questions, focused only on the global cognitive aspects of mental functions (Measso et al., 1993). The FAB is a brief, simple tool used to assess executive function (Dubois et al., 2000).

### Statistical analysis

Continuous variables were expressed as mean ± standard deviation (SD) and categorical variables as proportion (%). Statistical significance was set at *p*-value < 0.05, with 95% confidence intervals (CI). The characteristics of the population in terms of distributions and frequencies, means with standard deviations (SD) and medians were calculated. Linear regression models were applied to assess associations, using SVD and DVD at foveal, parafoveal and whole retina levels, of increased thicknesses (expressed as percentages) of the ganglionar cellular complex (GCC) and retinal nerve fiber layers (RNFL) as independent variables, with MMSE and FAB scores. We built three hierarchical nested models: an unadjusted model, a partially adjusted model (adjusted for age, gender, and education) and a fully-adjusted model [adjusted for age, gender and education Diabetes, Hypertension, SSI (lower) and visual acuity]. To reduce selection bias and simplify the reading of results we used a complete randomization algorithm for the eye selection, assigning the corresponding value (left or right eye) to the new variable thus created.

Statistical analyses were performed with RStudio software, Version 1.2.1335 using additional packages: tidyverse, kable.

## Results

From 2016 to 2019, 892 of the 1,929 participants in the Salus in Apulia Study underwent an ophthalmological examination including OCT and OCT-A scans. Of these, 318 subjects were excluded due to incomplete clinical evaluations, media opacity severely reducing visual acuity, macular diseases, glaucoma, hypertensive retinopathy, and erroneous scans including scans with segmentation failure. Overall, 574 older individuals (63% women) were eligible for the analysis presented in this study. Mean age of the whole sample was 73.82 ± 6.17 years. The average number of years of education was 6.94 ± 3.83 years. Mean MMSE and FAB scores were 26.16 ± 4.31 and 12.98 ± 3.82, respectively. All other sociodemographic and ophthalmological variables are reported in Table 1. Data of the whole Salus in Apulia sample are shown in Supplementary Table 1.

**TABLE 1:** Description of sociodemographic and variables of the whole sample. N:574.

	Mean ± SD	Median (min to max)
Age (years)	73.82 ± 6.17	73 (65 to 95)
BMI (Kg/m^2^)	27.83 ± 4.65	27.48 (18.36 to 47.65)
Gender		
Male	216 (37.60)	
Female	358 (63.40)	
Education (years)	6.94 ± 3.83	5 (0 to 18)
Hypertension	478 (83.4)	
Diabetes	48 (8.4)	
MMSE score	26.16 ± 4.31	28 (1 to 30)
FAB score	12.98 ± 3.82	14 (0 to 18)
BCVA RE (LogMAR)	0.13 ± 0.32	0.04 (0 to 1)
BCVA LE (LogMAR)	0.13 ± 0.3	0 (0 to 1)
IOP RE (mmHg)	14.45 ± 2.21	14 (10 to 20)
IOP LE (mmHg)	14.48 ± 2.22	15 (8 to 20)
GCC (μm)	96.01 ± 13.94	94.5 (44.75 to 237.85)
RNFL (μm)	95.77 ± 11.11	97 (62 to 128)
FAZ (mm^2^)	0.3 ± 0.26	0.28 (0.02 to 4.7)
Foveal SVD (%)	29.8 ± 6.84	29.33 (10.62 to 62.42)
Para foveal SVD (%)	51.94 ± 4.79	53.02 (31.28 to 60.26)
Whole retina SVD (%)	49.84 ± 4.36	50.7 (32.52 to 58.27)
Foveal DVD (%)	27.69 ± 7.66	26.84 (6.27 to 74.88)
Para foveal DVD (%)	57.11 ± 5.36	58.35 (28.18 to 66.13)
Whole retina DVD (%)	54.21 ± 5.01	55.29 (28.75 to 62.5)

All data are shown as mean ± sd, median (min to max) for continuous variables and as n (%) for proportions.

In the linear regression partially adjusted model, corrected for age, gender, and education (Table 2), foveal DVD (beta = 0.069, CI 95% 0.001–0.139) and whole DVD (beta = 0.064, CI 95% 0.001–0.127) showed a positive association with MMSE. Foveal SVD (beta = 0.081, CI 95% 0.019–0.142) and whole SVD (beta = 0.066, CI 95% 0.009–0.124) were also positively associated with the cognitive score. In the fully-adjusted model, the association remained significant only for foveal DVD (beta = 0.01, CI 95% 0.004–0.052), whole DVD (beta = 0.04, CI 95% 0.02–0.08), and whole SVD (beta = 0.04, CI 95% 0.02–0.07).

**TABLE 2:** Linear regression model on Mini-Mental State Examination (MMSE) as dependent variable and regressors.

	Unadjusted models			*Partially adjusted models			**Fully adjusted models		
	Coefficients	CI 95%	*P*-value	Coefficients	CI 95%	*P*-value	Coefficients	CI 95%	*P*-value
Foveal DVD	0.122	0.042–0.203	**<0.01**	0.069	0.001–0.139	0.05	0.01	0.004–0.052	**0.05**
Parafoveal DVD	–0.008	−0.06–0.044	0.76	0.007	−0.036–0.051	0.74	–0.02	−0.03–0.14	0.67
Whole DVD	0.11	0.036–0.183	**<0.01**	0.064	0.001–0.127	**0.04**	**0.04**	**0.02–0.08**	**0.02**
Foveal SVD	0.154	0.084–0.224	**<0.01**	0.081	0.019–0.142	**0.01**	0.02	−0.05–0.09	0.55
Parafoveal SVD	–0.015	−0.061–0.032	0.53	–0.005	−0.044–0.034	0.79	–0.04	−0.16–0.07	0.44
Whole SVD	0.12	0.062–0.193	**<0.01**	0.066	0.009–0.124	**0.02**	**0.04**	**0.02–0.07**	**0.02**
GCC	–0.005	−0.03–0.02	0.71	–0.002	−0.023–0.019	0.82	0.01	−0.02–0.04	0.99
RNFL	0.012	−0.021–0.044	0.48	–0.002	−0.029–0.025	0.87	–0.01	−0.04–0.04	0.91

*Corrected for age, gender, and education.

**Corrected for partially adjusted models regressors plus Diabetes, Hypertension, signal strength index (SSI) (lower) and visual acuity. Underlined and bold values indicate statistically significant data.

In addition, foveal SVD (beta = 0.01, CI 95% 0.003–0.05) and whole SVD (beta = 0.03, CI 95% 0.004–0.08) were positively associated with global cognitive function as assessed by the FAB (Table 3).

**TABLE 3:** Linear regression models on Frontal Assessment Battery (FAB) as dependent variable and regressor.

	Unadjusted models			*Partially adjusted models			**Fully adjusted models		
	Coefficients	CI 95%	*P*-value	Coefficients	CI 95%	*P*-value	Coefficients	CI 95%	*P*-value
Foveal DVD	0.08	0.008–0.152	**0.02**	0.035	−0.022–0.092	0.22	–0.04	−0.10–0.02	0.20
Parafoveal DVD	–0.02	−0.066–0.026	0.38	–0.008	−0.043–0.028	0.67	–0.03	−0.15–0.08	0.50
Whole DVD	0.073	0.007–0.138	**0.03**	0.036	−0.016–0.088	0.17	–0.06	−0.19–0.05	0.30
Foveal SVD	0.137	0.075–0.199	**<0.01**	0.071	0.021–0.121	**<0.01**	0.01	0.003–0.05	**0.03**
Parafoveal SVD	–0.021	−0.062–0.02	0.32	–0.013	−0.045–0.019	0.42	–0.02	−0.13–0.09	0.71
Whole SVD	0.113	0.054–0.171	**<0.01**	0.06	0.013–0.106	**0.01**	0.03	0.004–0.08	**0.04**
GCC	–0.005	−0.027–0.018	0.67	–0.001	−0.018–0.016	0.90	–0.01	−0.05–0.02	0.36
RNFL	0.009	−0.021–0.038	0.55	–0.002	−0.025–0.021	0.84	–0.03	−0.07–0.01	0.75

*Corrected for age, gender, and education.

**Corrected for partially adjusted models regressors plus Diabetes, Hypertension, signal strength index (SSI) (lower), and visual acuity. Underlined and bold values indicate statistically significant data.

We found no further significant association between the MMSE score or between the FAB score and the average thickness of the GCC and RNFL, and FAZ area.

## Discussion

In this large study of OCT-A findings in a Mediterranean population from Castellana Grotte aged over 65 years, we observed a direct linear association between the VD of the retinal capillary plexi, and global and frontal cognitive functions, measured with MMSE and FAB, respectively. A lower cognitive test score corresponded to a lower VD, particularly at the foveal site. The mechanism behind the retinal capillary density reduction in patients with cognitive impairment is not known. A reduction in the brain capillary density, both age-related (Bell and Ball, 1981; Vecino et al., 2016) and AD-related (Bell and Ball, 1981; Fischer et al., 1990; Buée et al., 1997; Vecino et al., 2016), has been demonstrated in several studies.

The anti-angiogenic activity of perivascular β-amyloid plaques accumulation has been proposed as a cause of capillary density reduction in patients with AD (Paris et al., 2004a,b). In particular, decreased angiogenesis, due to sequestration of vascular-endothelial growth factor (VEGF) in β-amyloid plaques and competitive binding of β-amyloid to VEGF receptor 2, has been proposed as the possible mechanism underlying cerebral VD reductions (Yang et al., 2004).

In addition, an age-related reduction in angiogenesis capacity was reported (Black et al., 1989; Rivard et al., 1999). With aging, the expression of hypoxia-inducible factor-1 (HIF-1) is reduced (Rivard et al., 2000; Chavez and LaManna, 2003). HIF-1 is the transcription factor leading to the synthesis of VEGF in hypoxia conditions (Saint-Geniez and D’Amore, 2004). The reduction in HIF-1 is associated with a reduction in VEGF and neuronal loss (Rapino et al., 2005). With aging in AD, therefore, vascular recovery from hypoxia is impaired.

Furthermore, direct β-amyloid endothelial damage, causing small vessels destruction, has also been noted (Li et al., 2020). Koronyo-Hamaoui et al. (2011) identified postmortem β-amyloid plaques in the retina of AD patients. In a histological study of patients with AD, β-amyloid plaques were found, associated with retinal blood vessels and located in the perivascular area (Koronyo et al., 2017). Although in the classical hypothesis β-amyloid plaques and neurofibrillary tangles are recognized as the main pathogenic mechanism, different studies have attributed an increasing importance to the various vascular alterations present in patients with AD and cognitive impairment (Cheung et al., 2014; Feke et al., 2015; Sweeney et al., 2019).

Chronic cerebral hypoperfusion induces a reduction of nutrients supply to the brain, causing direct damage not only to parenchymal cells but also to the vascular constituents of the blood-brain-barrier (BBB) (Sweeney et al., 2019). BBB dysfunction mediates the indirect neurotoxic effect by promoting oxidative stress, inflammation paracellular permeability, and dysregulation of nitric oxide, a key regulator of regional blood flow (Sweeney et al., 2019). All these events trigger a vicious circle in which cerebral perfusion is reduced and the neurodegenerative process is accelerated. Reciprocal interactions between vascular dysfunction and neurodegeneration could further contribute to the development of the disease (Sweeney et al., 2019). Thus, the previously observed (Bulut et al., 2018; Yoon et al., 2019; Chua et al., 2020; Criscuolo et al., 2020; Wu et al., 2020; Hui et al., 2021; Zhang et al., 2021) close link between microvascular alterations and cognitive impairment, also confirmed by our results, shows that retinal VD may be an ocular biomarker of age-related neurocognitive disease.

Conversely, we did not find any association between GCC and RNFL thickness and MMSE and FAB scores. Several studies have revealed thinning of RNFL in MCI and AD patients as compared to controls (Berisha et al., 2007; Paquet et al., 2007; Kesler et al., 2011), and a linear association between RNFL volume reduction and neocortical Aβ accumulation, after controlling for normal aging (Santos et al., 2018).

However, it should be considered that RNFL thickness in healthy old adults is extremely heterogeneous (Poinoosawmy et al., 1997; Parikh et al., 2007). While some studies were unable to find differences in retinal layers thickness between MCI patients and controls (Cheung et al., 2015; Feke et al., 2015; Pillai et al., 2016; Golzan et al., 2017; Kwon et al., 2017), others found equal values of RNFL thickness between MCI patients and healthy subjects (Oktem et al., 2015; Ferrari et al., 2017). Furthermore, thinning of the RNFL is also present in other types of dementia, such as frontotemporal dementia (Ferrari et al., 2017), dementia with Lewy bodies, dementia associated with Parkinson’s disease (Moreno-Ramos et al., 2013) and with cerebral autosomal dominant arteriopathy with subcortical infarcts and leuco-encephalopathy (CADASIL) (Parisi et al., 2007), challenging the specificity of OCT findings in cognitive impairment.

The strength of this study lies in the large number of subjects analyzed. We also adjusted for age, gender, and education during analysis, and had excluded confounding factors such as diabetes, ocular disease, and media opacity reducing visual acuity so much to interfere with OCT image acquisition and analysis. Furthermore, the measurements of one eye were randomly adopted for each subject as a good practice for statistical analysis (Armstrong, 2013).

A limitation of the study is that we did not evaluate the axial length of the eyes analyzed with OCT-A. Extreme axial length can cause an alteration in the retinal VD calculation (Yang et al., 2016). However, the large study population and the exclusion of patients with extreme refractive defects should have reduced this possible bias.

A methodology limit of the present study is the cross-sectional nature of the data, preventing assessment of the direction of the association, and introducing a high risk of reverse causality bias. An important methodological point is that clearly, we could not adjust for differences in prior cognitive ability when analyzing the association between retinal parameters and cognitive ability later in life. Childhood cognitive ability accounts for a large proportion of variance in cognitive ability in old age (Deary et al., 2004). Differences in prior cognitive ability are associated with health, morbidity and mortality outcomes, including those related to cerebrovascular disease (Gottfredson and Deary, 2004; Deary et al., 2010). Hence, the cross-sectional association between retinal vascular measures and cognitive ability reported in this study could inevitably be confounded by prior cognitive ability. Lastly, with did not correct the models for cardiovascular and cerebrovascular diseases due to the lack of these data.

In conclusion, the present findings confirmed the association between impaired cognitive test scores and retinal VD in older subjects, suggesting that this may be a potential ocular biomarker of age related-neurocognitive disease and confirming the OCT-A as a non-invasive tool to identify this biomarker in preclinical stages of cognitive impairment. Further larger studies, in longitudinal cohorts or with a randomized clinical trial design, are needed to test the effectiveness of applying retinal capillary density as a novel biomarker to predict the incidence and progression of cognitive impairment, also at population level.

## Data availability statement

The raw data supporting the conclusions of this article will be made available by the authors, without undue reservation.

## Ethics statement

The studies involving human participants were reviewed and approved by Institutional Review Board of the “National Institute of Gastroenterology “S. De Bellis” (Approval Code: 68/CE De Bellis; Approval Date: April 9, 2019). The patients/participants provided their written informed consent to participate in this study.

## Author contributions

GG and GSb: conceptualization. GG, AN, PP, AP, VP, RB, RG, MB, FCass, AC, LLan, GSc, MG, AG, RSe, and MS: data curation. FCast: formal analysis. RSa: funding acquisition and project administration. GG and AN: investigation. GG, AN, and RSa: methodology. GG, GSb, and AN: resources. GSb and RSa: supervision. GG and AN: writing – original draft preparation. GA, RSa, LLam, and FB: writing – review and editing. All authors have read and agreed to the published version of the manuscript.

## Footnotes

1
http://www.stard-statement.org/

2
https://www.strobe-statement.org/
