# Supplementary material for: Correlation between retinal vessel rarefaction and psychometric measures in an older Southern Italian population
Source: Front Aging Neurosci. 2022 Sep 23;14:999796. doi: 10.3389/fnagi.2022.999796 (PMC9541429; doi:10.3389/fnagi.2022.999796)
Supplement: Supplementary file 1 [file Data_Sheet_1.docx]

Supplementary Material

# Supplementary Figures

**S1 Fig.** **Optical coherence tomography angiographic (OCT-A) image of the foveal avascular zone (FAZ) areas at the deep vascular plexus.**


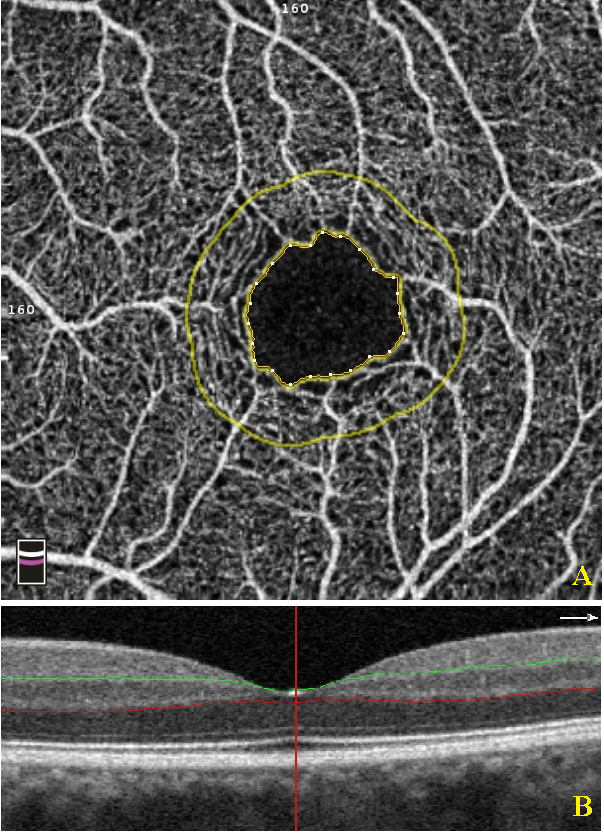


The FAZ area border (inner yellow line) was outlined automatically, and the surface area was then measured in square millimeters using built-in software under the flow option in the deep vascular plexus (A) The colored lines (red and green) in horizontal OCT B-scans show segmentation lines defining the depths in the retinal tissue (B).

**S2 Fig.** **Optical coherence tomography images of the ganglion cell complex (GCC) and retinal nerve fiber layer (RNFL) thickness.**


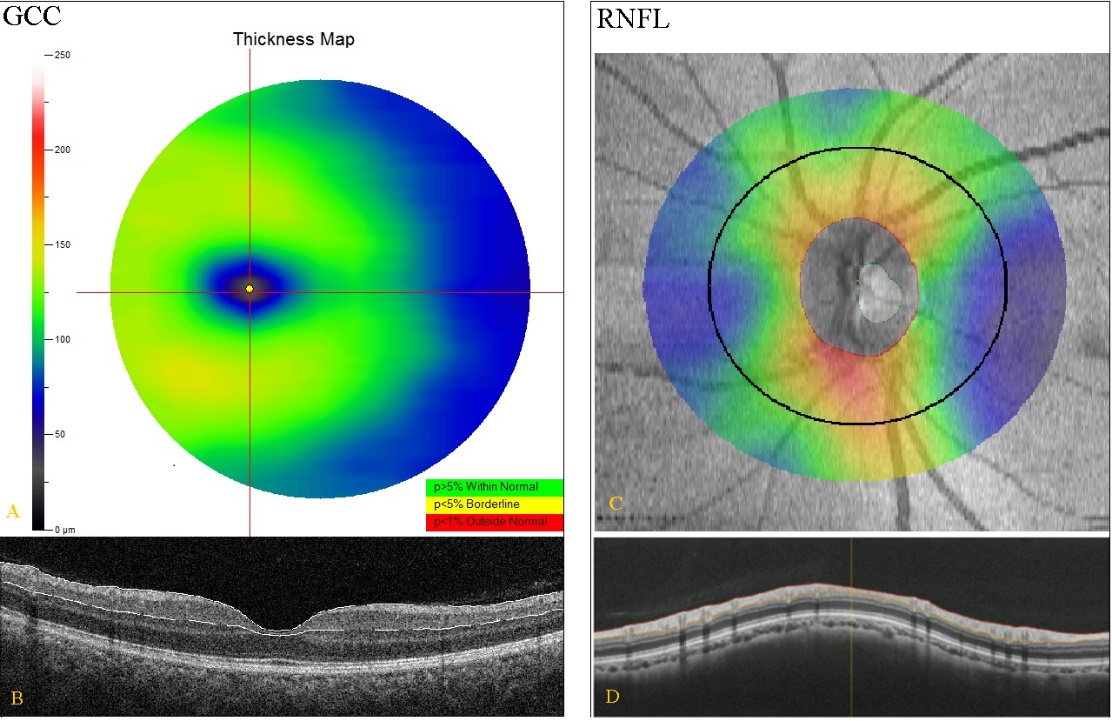


The device measures GCC and RNFL thickness within an automatically rendered 7-mm2 area, centered 1 mm temporally to the fovea. The system produces a color-coded thickness map for interpretation. (A,C) Thicker regions of GCC and RNFL are displayed as yellow and orange, whereas thinner regions are displayed as blue and green. Acquired thicknesses are compared with values from a normative database and displayed as a significance map. The color-coded map shows the corresponding probabilities of deviation from the normal range, based on comparison with an age-matched control group of healthy subjects. (B,D) Cross-sectional B-scan displays the segmentation used for the GCC and RNFL analyses. (B) The traced boundaries for the GCC scan (white lines) include the inner limiting membrane and the outer IPL. (D) The traced boundaries for the RNFL scan (colored lines) include the inner limiting membrane and the outer IPL.

**Table S1: Description of Sociodemographic Characteristics of general population sample. N: 1929**

|  | mean ± sd | median  (min to max) |
| --- | --- | --- |
| Age (years) | 73.56 ± 6.3 | 72 (65 to 95) |
| FAB score | 13.06 ± 3.79 | 14 (0 to 18) |
| MMSE | 26.3 ± 4.26 | 28 (1 to 30) |
| Educational Level (years) | 6.94 ± 3.83 | 5 (0 to 23) |
